# Lack of Blue Light Regulation of Antioxidants and Chilling Tolerance in Basil

**DOI:** 10.3389/fpls.2022.852654

**Published:** 2022-04-07

**Authors:** Dorthe H. Larsen, Hua Li, Samikshya Shrestha, Julian C. Verdonk, Celine C. S. Nicole, Leo F. M. Marcelis, Ernst J. Woltering

**Affiliations:** ^1^Horticulture and Product Physiology Group, Wageningen University and Research, Wageningen, Netherlands; ^2^Signify Research Laboratories, Eindhoven, Netherlands; ^3^Food and Biobased Research, Wageningen University and Research, Wageningen, Netherlands

**Keywords:** blue light, basil, vertical farming, anthocyanins, antioxidants, chilling injury, quality

## Abstract

Blue light, measuring from 400 to 500 nm, is generally assumed to increase the content of antioxidants in plants independent of the species. Blue light stimulates the biosynthesis of phenolic compounds such as flavonoids and their subclass anthocyanins from the phenylpropanoid pathway. Flavonoids, anthocyanins, and phenolic acids are strong reactive oxygen species (ROS) scavengers and may lessen the symptoms of abiotic stresses such as chilling. We tested the hypothesis that a high percentage of blue light induces the accumulation of antioxidants and that this effect depends on the photosynthetic photon flux density (PPFD, 400–700 nm). The effect may be more pronounced at a lower PPFD. We investigated the changes in primary and secondary metabolites of basil in response to the percentage of blue light (9, 33, 65, and 100%) applied either as a 5-day End-Of-Production (EOP) treatment or continuous throughout the growth cycle in the green cv. Dolly. We also studied if the response to the percentage of blue light (9 or 90%) was dependent on the total PPFD (100 or 300 μmol m^–2^ s^–1^ PPFD) when applied as a 5-day EOP treatment in the green cv. Dolly and the purple cv. Rosie. For both green and purple basil, it was found that the percentage of blue light had little effect on the levels of antioxidants (rosmarinic acid, total ascorbic acid, total flavonoids, and total anthocyanins) at harvest and no interactive effect with PPFD was found. Antioxidants generally decreased during postharvest storage, wherein the decrease was more pronounced at 4 than at 12°C. Chilling injury, as judged from a decrease in F_*v*_/F_*m*_ values and from the occurrence of black necrotic areas, was not affected by the percentage of blue light. Particularly, chilling tolerance in the purple cultivar was increased in plants grown under higher PPFD. This may be related to the increased levels of soluble sugar and starch in leaves from high PPFD treated plants.

## Introduction

Basil (*Ocimum basilicum* L.) is rich in antioxidants, in particular, polyphenolic compounds from the phenylpropanoid pathway such as rosmarinic and chicoric acid (Kwee and Niemeyer, 2011). Basil also contains compounds from the flavonoid (sub) family such as quercetin, rutin, and kaempferol. Mostly basil exists as green varieties but some varieties are purple due to anthocyanins which are a subgroup of flavonoids (McCance et al., 2016). Compounds such as anthocyanins, flavonoids, and phenolic acids have strong antioxidant capacity. Antioxidants can scavenge reactive oxygen species (ROS) and protect the plants from oxidative damage thus contributing to tolerance against abiotic stress such as chilling and drought (Ahmed et al., 2014). Chilling injury occurs in basil when it is exposed to temperatures below 10–12°C during growth, storage, or transport resulting in the development of dark necrotic spots (Lange and Cameron, 1994). During chilling, a cascade of events occurs: the lipid bilayer in the cell membranes can go from a flexible to a solid gel state which may result in membrane malfunction, ion leakage, and excessive formation of ROS. ROS will further lead to the damage of the DNA, membrane lipids, and proteins, thereby being severely damaging to the plant (Sevillano et al., 2009). Antioxidants, such as phenolic compounds, can counteract ROS and ameliorate chilling tolerance (Das and Roychoudhury, 2014). Increasing the content of antioxidants such as flavonoids and anthocyanins may improve the tolerance to chilling temperatures. In addition, an increase in sugars, starch, and antioxidants is beneficial for consumers as it improves the products’ nutritional value. In the production phase, antioxidants can be increased through the modulation of the growth environment (Larsen et al., 2022). Such modulation can be facilitated by light-emitting diodes (LEDs) through which we can easily increase both the light intensity and change the light spectrum. LEDs are particularly used in greenhouses and in vertical farming (SharathKumar et al., 2020). Light intensity and spectrum can affect the content of phenolic acids, flavonoids, and anthocyanins. In particular, blue light (400–500 nm) has been found to stimulate the biosynthesis of compounds from the phenylpropanoid pathway such as flavonoid and anthocyanin content in several crops: in fruit and leaves of strawberry (Piovene et al., 2015; Zhang et al., 2018), lettuce (Samuoliene et al., 2013), and Arabidopsis (Chen et al., 2006). Blue light has also been found to stimulate the biosynthesis of rosmarinic acid, chicoric acid, chlorogenic acid, *p*-OH-cinnamic acid derivative, 2-*O*-feruloyl tartaric acid, and quercetin rhamnoside in green basil (Taulavuori et al., 2013, 2016), and phenolic acids in red lettuce (Ouzounis et al., 2015). In addition, blue light increased the content of vitamin C in pak choi with a photosynthetic photon flux density (PPFD, 400–700 nm) up until 100 μmol m^–2^ s^–1^ after which it decreased at a higher PPFD (Zheng et al., 2018). Although blue light has been widely accepted to stimulate the biosynthesis of compounds from the flavonoid branch of the phenylpropanoid pathway it is not fully understood why compounds such as vitamin C should increase. The energy content of a blue light photon is higher than its red counterparts due to blue light having a lower wavelength than red. Thus, blue photons might result in a stress reaction.

Application of increased light intensity during the last phase of the growth as an End-Of-Production (EOP) treatment showed to be sufficient to increase the content of secondary metabolites without having adverse effects on plant morphology (Gomez and Jimenez, 2020; Larsen et al., 2020, 2022; Min et al., 2021). In red lettuce, an EOP treatment with 69% blue light has increased anthocyanins but not flavonoids (Gomez and Jimenez, 2020). However, a change in the spectrum as EOP treatment is yet to be studied in basil. We hypothesized that an increased percentage of blue light would increase the content of antioxidants, such as phenolic acids, flavonoids, and anthocyanins, thereby improving chilling tolerance. Furthermore, we hypothesized that the effect of a high percentage of blue light might have an interactive effect on the PPFD (400–700 nm). Spectral effects may be less on the accumulation of antioxidants under higher PPFD as the PPFD might dominate the overall plant response.

First, we investigated the changes in primary and secondary metabolites of basil in response to the percentage of blue light (400–500 nm) in the spectrum applied either as an EOP treatment or continuous throughout the growth cycle. Second, we studied if the light intensity interacts with the percentage of blue light applied as EOP treatment in a green and purple cultivar and further if this improves the postharvest chilling tolerance.

## Materials and Methods

### Experimental Set-Up

For this study, two cultivars of basil (*Ocimum basilicum* L.), cv. Dolly (green leaves) and cv. Rosie (purple leaves that are rich in anthocyanin) (Enza Zaden, Enkhuizen, the Netherlands) were grown in a climate chamber. Plants were grown according to Larsen et al. (2020, 2022). The seeds were sown as single seeds in stone wool plugs in trays of 240 plugs (Grodan Rockwool B.V., Roermond, the Netherlands). The most morphologically similar plants were transplanted to 7.5 cm × 7.5 cm × 6.5 cm stone wool blocks (Grodan Rockwool B.V., Roermond, the Netherlands) after 15 days. For the growth of the plants, a vertical farming set-up was used. Each compartment had a dimension of 0.8 m × 1.3 m × 1 m, (w × l × h) and a planting density of 123 plants m^–2^. The two cvs were grown in different compartments to maintain a similar PPFD at the top of the plants. Throughout the experiments, the heights of the light frames were adjusted to maintain the desired PPFD. The light frames were kept 25 cm above the plants. In the climate chamber. the day/night temperature was set at 25°C, the relative humidity at 75%, and carbon dioxide (CO_2_) was kept at ambient concentrations. The temperature and relative humidity deviated within ± 10% (RH) and 1°C (T) from the setpoints and were logged with KeyTag dataloggers (KTL-508, KeyTag, Leiderdorp, the Netherlands).

Plants were watered through an ebb and flow system. At all growth stages, plants were kept well-watered. Plants were watered with a nutrient solution of pH 5.7, EC 1.7 dS m^–1^, 8.5 mM NO_3_^–^, 3.9 mM SO_4_^2–^, 1.5 mM HPO_4_^2–^, 1.5 mM NH_4_^+^, 5.5 mM K^+^, Ca^2+^ 4 mM, 1.5 mM Mg^2+^, 0.2 mM Cl^–^, 30 μM Fe^3+^/Fe^2+^, 5 μM Mn^2+^, 5 μM Zn^2+^, 35 μM H_2_BO_3_^–^, 1 μM Cu^+^/Cu^2+^, and 1 μM MoO_4_^2–^ before transplanting. After transplanting, the EC measured 2.3 dS m^–1^ and the concentration of the nutrients was raised correspondingly.

The response to the light treatments on plant growth and morphology (i.e., plant height, leaf area, and fresh and dry mass at harvest) from these experiments were described by Larsen et al. (2020).

#### Blue Light Duration (Experiment 1)

In Experiment 1, we investigated the response of cv. Dolly to different percentages of blue light applied either as a continuous treatment throughout the growth (i.e., for 25 days) or as EOP treatment during the last 5 days before harvest. Seedlings grew under red-white light from LEDs (Green Power LED production module, 120 cm, Philips Eindhoven, the Netherlands) with a PPFD of 150 μmol m^–2^ s^–1^. The red-white light contained 9% blue (B) (400–500 nm), 19% green (G) (500–600 nm), and 70% red (R) (600–700 nm), as well as 1% far-red (FR) (700–800 nm) lights. For the blue light treatments, the different percentages of blue light were made by using pure blue (Green Power LED production module, 120 cm, Blue, Philips Eindhoven, the Netherlands), (Green Power LED research module, Blue, Philips Eindhoven, the Netherlands) and red-white LEDs. When the plants were transplanted, they were treated with four different blue light treatments for 25 days with a total PPFD of 300 μmol m^–2^ s^–1^ (Table 1.). In addition, plants were grown under red-white light (PPFD, 300 μmol m^–2^ s^–1^) in three other treatments for 20 days after which they were treated with different blue light treatments for 5 days (Table 1). For all light treatments, the spectral intensity was measured with a spectroradiometer (USB2000 spectrometer, Ocean Optics, Duiven, 110 Netherlands). Throughout the experiment, the day length was 16 h. This whole experiment with a similar set-up was conducted 2 times.

**TABLE 1:** Photosynthetic photon flux density (PPFD) (400–700 nm) and spectra of the treatments for Experiments 1 and 2.

Treatments	Treatment duration (days)	PPFD (μmol m^–2^ s^–1^)	Blue light (%)	Green light (%)	Red light (%)	Far-red light (%)
**Exp. 1**						
9%	25 and 5	300	9	19	70	1
33%	25 and 5	300	33	14	51	1
65%	25 and 5	300	65	7	26	0
100%	25 and 5	300	100	0	0	0
**Exp. 2**						
Low PPFD, low blue	5	100	9	19	70	1
Low PPFD, high blue	5	100	90	2	8	0
High PPFD, low blue	5	300	9	19	70	1
High PPFD, high blue	5	300	90	2	8	0

*Percentages of the spectra; blue light (400–500 nm), green light (500–600 nm), red light (600–700 nm), far-red light (700–800 nm) are expressed as percentages of the total photon flux density (400–800 nm).*

#### Blue Light and the Interactive Effect With PPFD (Experiment 2)

In Experiment 2, we investigated the response of cultivars Rosie (purple) and Dolly (green) to EOP treatments with an increased percentage of blue light. We also investigated the interaction with PPFD during the last 5 days before harvest. Seedlings grew under 200 μmol m^–2^ s^–1^ red-white LED light. Plants were transplanted and continued to grow for another 15 days under 200 μmol m^–2^ s^–1^ red-white light. The last 5 days before harvest plants were treated with EOP treatments with either a low (100 μmol m^–2^ s^–^1) or high (300 μmol m^–2^ s^–1^) PPFD, in combination with a low (9%) and high (90%) percentage of blue light (Table 1). For the blue light treatments, the different percentages of blue light were made by using two types of pure blue LEDs (Green Power LED production module, 120 cm, Blue, Philips Eindhoven, the Netherlands) (Green Power LED research module, Blue, Philips Eindhoven, the Netherlands) and red-white LEDs. For all light treatments, the spectral intensity was measured with a spectroradiometer (USB2000 spectrometer, Ocean Optics, Duiven, 110 Netherlands). Throughout the growth, the day length was 18 h. This whole experiment with a similar set-up was conducted 3 times for cv. Dolly and 4 times for cv. Rosie.

#### Postharvest Storage and Sampling

The plants were harvested 40 (Experiment 1) or 35 (Experiment 2) days after sowing. The border plants were excluded from the sampling. The postharvest storage was done according to Larsen et al. (2022). For postharvest storage and sampling, three-leaf pairs were taken per plant. The oldest and youngest underdeveloped leaves were excluded. The leaves were stored in plastic boxes (16 cm × 11 cm × 6 cm), which combined leaves from two plants per box. The wetted filter paper was added to the bottom of the boxes to keep the humidity high. For the leaves to avoid direct contact with the wet filter paper, a small piece of plastic was added on top of it. The leaves from the two plants were separated by a piece of plastic. To avoid the build-up of CO_2_ or ethylene, nine holes were made in the lids with a 1 mm syringe needle. During storage, the boxes were randomized in a cold cabinet in darkness at 4 or 12°C. In the boxes, the temperature and relative humidity deviated within ± 2% (RH) and 0.3°C (T) from the setpoints and were recorded with KeyTag dataloggers (KTL-508, KeyTag, Leiderdorp, the Netherlands).

In Experiment 1, measurement and sampling were done on day 0 (at harvest) and 5, 10, and 15 days after harvest for EOP treated plants. In Experiment 2, measurements and sampling were done on day 0 (day of harvest), while days 3 and 6 for cv. Dolly and for cv. Rosie the sampling continued on days 9 and 12. Two postharvest storage boxes (i.e., each containing leaves from two individual plants per block per light treatment) were sampled on each sampling day.

During sampling, an overall visual quality score was given to the leaves of each sampled plant to determine the chilling injury level. In Experiment 2, the maximum quantum yield of PSII (F_*v*_/F_*m*_) was measured in addition to the scoring. Following the scoring and measuring of F_*v*_/F_*m*_, the leaves were frozen in liquid nitrogen and ground with an IKA-A 11 basic analytical mill (im-lab, Boutersem, Belgium). Samples were stored at −80°C for further analysis of metabolite content. Each sample consisted of leaves derived from 4 plants.

### Carbohydrates

Carbohydrates were measured according to Larsen et al. (2022). Briefly, 300 mg of frozen ground leaves were extracted with 5 ml of 85% ethanol in a shaking water bath at 80°C for 20 min. Samples were centrifuged for 5 min at 8,500 RCF (Universal 320R, Hettich, Sigma-Aldrich, Darmstadt, Germany) and 1 ml of the supernatant was dried with a vacuum centrifuge (Savant SpeedVac SPD2010, Thermo Fisher Scientific, Waltham, MA, United States) for 120 min at 50°C and 5.1 mbar. The remaining pellet with supernatant was later used for starch determination.

The dried samples were re-suspended in 2 ml of 0.01 N hydrochloric acid and sonicated for 10 min (Branson 2800, Richmond, VA, United States). The samples were vortexed and centrifuged at 21,100 RCF for 5 min (Sorvall Legend Micro 21R, Thermo Fisher Scientific, Waltham, MA, United States).

Amino acids and other amino compounds were removed from the sample solution by trapping with a SPE column (UCT CLEAN-UP BCX columns, BGB analytik Benelux B.V. Harderwijk, The Nethelands, 100 mg/1 ml), eluted with 0.01 N hydrochloric acid.

The samples were diluted ten times and glucose, fructose, and sucrose were quantified using High-Performance Anion Exchange Chromatography with Pulsed Amperometric Detection (HPAEC-PAD; Dionex ICS5000, Thermo Fisher Scientific, Waltham, MA, United States), with a CarboPac1 column (250 mm × 2 mm, Thermo Fisher Scientific, Waltham, MA, United States) and eluted with 100 mM NaOH at a flow rate of 0.25 ml/min at 25°C.

For starch determination, the stored pellet was used. The pellet was washed three times with 80% ethanol, dried for 20 min in a vacuum centrifuge at 50°C and 5.1 mbar. For the resuspension of the dried pellet, 2 ml of 1 g/L thermostable alpha-amylase (SERVA Electrophoresis GmbH, Heidelberg, Germany) was used. The samples were incubated at 90°C for 30 min. Before further incubation at 60°C for 15 min, 1 ml of 0.5 g/L amyloglucosidases (10115 Sigma-Aldrich, Darmstadt, Germany) in 50 mM citrate buffer (pH 4.6) was added to the samples. The samples were centrifuged at 21,100 RCF for 5 min and diluted 50–100 times. Glucose was quantified using HPEAC-PAD (see description above).

A conversion factor was made for each sample to convert them from fresh weight to dry weight. In brief, 400 ± 40 mg of fresh frozen was weighed into a reaction tube and oven-dried for 8 h at 70°C. Data was expressed on the base of dry weight as mg/g DW.

### Rosmarinic and Chicoric Acid

Phenolic acids were extracted according to Larsen et al. (2022). Briefly, 250 ± 20 mg of frozen ground leaves were extracted with 1.5 ml of 80% methanol with 2.5% formic acid for 15 min in an ultrasonic bath (Branson 2800, Richmond, VA, United States). The supernatant was filtered through a cellulose syringe filter 0.45 μm, and analyzed according to the method of Kwee and Niemeyer (2011), with modifications. In Experiment 1, samples were measured on an HPLC system (Waters, Knowloon, Hongkong) with a UV dual-wavelength detector and autosampler and a Vydac 201TP54 (C18, 5 μm, 300 Å, 4.6 mm × 250 mm) reverse-phase (RP) column. In Experiment 2, samples were measured on an HPLC system with a GS50 pump (Dionex, Thermo Fisher Scientific, Waltham, MA, United States), a 340S UV-VIS detector (Dionex, Thermo Fisher Scientific, Waltham, MA, United States), and a MIDAS autosampler (Spark, Emmen, the Netherlands) using a LiChrospher 100 RP-18 (5 μm), 150 × 4 mm column (Merck, Amsterdam, the Netherlands). Samples were eluted with 2.5% formic acid in H_2_O (A) and acetonitril (B) with a linear gradient of: 85% A, 0 min; 75% A, 6 min; 0% A, 8.5 min [0% A, 9 min; 85% A, 11.5 min; 85% A, 14 min]. Analytes were detected at 330 nm.

For quantification, calibration curves were prepared with standards (Extrasynthese, Genay, France) from 0 to 500 mg/L. Data were expressed on the base of dry weight as mg/g DW.

### Total Ascorbic Acid

Ascorbic acid (AsA) was measured according to Min et al. (2021). Total AsA (TAsA) is a large antioxidant group in leafy vegetables, also defined as vitamin C (Min et al., 2021). TAsA is the sum of AsA and dehydroascorbic acid (DHA). Extraction of AsA was done from 200 mg frozen ground leaves with 1 ml 3.3% meta-phosphoric acid (MPA) and sonicated (Branson 2800, Richmond, VA, United States) for 10 min in darkness at 0°C. The samples were centrifuged at 21,100 RCF (Sorvall Legend Micro 21R, Thermo Fisher Scientific, Waltham, MA, United States) for 10 min at 4°C. For analysis of AsA, the supernatant was filtered through a cellulose syringe filter of 0.45 μm of cellulose into an amber HPLC vial. Furthermore, for TAsA analysis, 100 μl of the filtered extract was transferred to another HPLC vial and 50 μl of 5 mM dithiothreitol in 400 mM Tris base was added. To convert DHA to AsA, the vials were kept in darkness at room temperature. The reaction was stopped after 15 min by adding 50 μl 8.5% o-phosphoric acid. AsA was measured on an HPLC consisting of a GS50 pump (Dionex, Thermo Fisher Scientific, Waltham, MA, United States), a 340S UV-VIS detector (Dionex, Thermo Fisher Scientific, Waltham, MA, United States) with a MIDAS autosampler (Spark, Emmen, the Netherlands), and a ProntoSIL 120-3 C18 AQ (250 × 3 mm column) (Knauer, Berlin, Germany). For the elution of column, 400 μL/L H3PO4 + 2.5 ml/L MeOH + 0.1 mM EDTA in H_2_O was used with a wash step consisting of 30% acetonitrile in H_2_O at a flow rate of 0.35 ml/min at 35°C. The detection of AsA was done at 243 nm. A standard with AsA in 3.3% MPA was used for calibration. The amount of TAsA was calculated as the sum of the AsA and the AsA converted from DHA. Data was expressed on the base of dry weight as mg/g DW.

### Total Anthocyanin Content

Total anthocyanin content was extracted from 300 mg frozen ground basil tissue with 1.5 ml 50% MeOH along with 1% formic acid in an ultrasonic bath (Branson 2800, Richmond, VA, United States) for 15 min. Samples were centrifuged at 15,000 RCF (Sorvall Legend Micro 21R, Thermo Fisher Scientific, Waltham, MA, United States) at 4°C for 15 min. The supernatant was filtered through a 0.45 μm cellulose filter. Samples were diluted 5 times and measured in a cuvette at wavelength of λ = 530 nm in a spectrophotometer (Genesys 50, Thermo Fisher Scientific, Waltham, MA, United States) against a blank. The total content of anthocyanins was expressed as mg/g with cyanidin chloride as standard in the range 1–25 mg/L.

### Total Flavonoid Content

Total flavonoid content was determined by aluminum chloride colorimetric assay (Zhishen et al., 1999). Total flavonoid content was extracted from 300 mg of frozen ground basil and 1.5 ml of methanol/H_2_O_/_acetone (60:30:10 v/v/v) in an ultrasonic bath (Branson 2800, Richmond, VA, United States) for 15 min. Samples were centrifuged at 15,000 RCF (Sorvall Legend Micro 21R, Thermo Fisher Scientific, Waltham, MA, United States) at 4°C for 10 min, and the supernatant was collected. Catechin was used as a quantifying standard. In a 3-ml cuvette, 50 μL of the extracted sample was mixed with 1.95 ml water and 75 μL of 5% NaNO_2_. After 6 min 150 μL of 10% AlCl_3_ was added and after another 5 min, 500 μL of 1 M NaOH was added. The absorbance was measured at a wavelength of λ = 250 nm in a spectrophotometer (Genesys 50, Thermo Fisher Scientific, Waltham, MA, United States) against a blank. Data was expressed on the base of dry weight as mg/g DW.

### Hydrogen Peroxide

Hydrogen peroxide (H_2_O_2_) was determined according to Junglee et al. (2014) with some modifications. H_2_O_2_ was extracted from 0.1 g of frozen ground basil leaves with 0.4 ml of 0.1% TCA, 0.4 ml of potassium phosphate buffer (pH 7.6), and 0.8 ml of potassium iodide. After incubation for 10 min at 4°C, the samples were centrifuged at 15,000 RCF (Sorvall Legend Micro 21R, Thermo Fisher Scientific, Waltham, MA, United States) at 4°C for 10 min, and the supernatant was collected. Samples were measured in UV-cuvettes at a wavelength of λ = 350 nm in a spectrophotometer (Genesys 50, Thermo Fisher Scientific, Waltham, MA, United States) against the blank. For quantification, a calibration curve was prepared with H_2_O_2_ solutions with concentrations from 10 to 400 μmol/L. For each sample, three technical replicates were prepared. Data were expressed on the base of dry weight as mg/g DW.

### Maximum Chlorophyll Fluorescence

Chilling injury was measured as an F_*v*_/F_*m*_ ratio previously described by Larsen et al. (2022). F_*v*_/F_*m*_ is the maximum quantum yield of the primary photochemical reactions or PSII in dark-adapted leaves. Per stored box, containing leaves from two plants, one leaf from the upper leaf-pair and one leaf from the middle leaf pair per plant were measured. First leaves were dark-adapted at 20°C for 20 min after which the measurement of chlorophyll fluorescence was done using a PSI closed Fluorcam 800-C chlorophyll fluorescence imaging system (PSI, Drasov, Czech Republic). To operate the fluorcam and analyze the images, the Fluorcam software version 7 was used, according to the method of Hogewoning and Harbinson (2007).

### Overall Visual Quality

Overall visual quality (OVQ) was evaluated using a scoring system according to Larsen et al. (2022). The scores were given based on visual symptoms associated with chilling injury and general symptoms appearing at non-chilling temperatures. A score between 1 and 8 was given based on the visual symptoms (i.e., 1 being the worst and 8 being the best). The consumer acceptance limit was set at the score of 5, which represented the end of shelf life. The scores would be reduced due to symptoms such as dark spots/discoloration, fungal appearance, degree of crispness, degree of wilting, leaf shininess, and presence of characteristic curved leaf shape (Supplementary Table 1).

### Statistical Setup and Analysis

The experiments were carried out in a complete randomized block design. The light treatments for the different cultivars in either the green cv. Dolly or the purple cv. Rosie were located in separate compartments. Experiment 1 was carried out two times (2 blocks) and Experiment 2 was carried out three times (3 blocks) for cv. Dolly and four times for cv. Rosie (4 blocks). The border plants were excluded from the analysis. For the chemical analysis at harvest, four replicate plants were sampled per light treatment in each block. The rest of the plants were stored for postharvest sampling. For postharvest storage at 4 and 12°C, the leaves from two plants were packed in one plastic box (see description above). Two boxes (i.e., leaves from four plants) per cv. and light treatment were sampled for overall visual quality and chemical analysis per postharvest timepoint. As one replicate an average value of each block was used for further statistical analysis. For each block, the chemical analysis was done on leaves from four plants as a pooled sample. The means are based on the number of blocks x four replicate plants.

The data were analyzed with Genstat (VSN International, 19th Edition). The assumptions of homogeneity and normality of the residuals were tested with Bartlett’s test and the Shapiro-Wilk test. In the case that the data did not follow the assumption, the data were transformed with the natural logarithm, after which it followed the assumption. Thereafter, the data were analyzed using a two-way ANOVA per time point and storage temperature with the *post hoc* test Fisher’s protected LSD. For Experiment 1, the test was conducted with a probability level of α = 0.1 because the experiment only had two blocks, while for Experiment 2, the probability level was α = 0.05

## Results

### Metabolite Content in Response to Percentage and Duration of Blue Light

**FIGURE 1:**
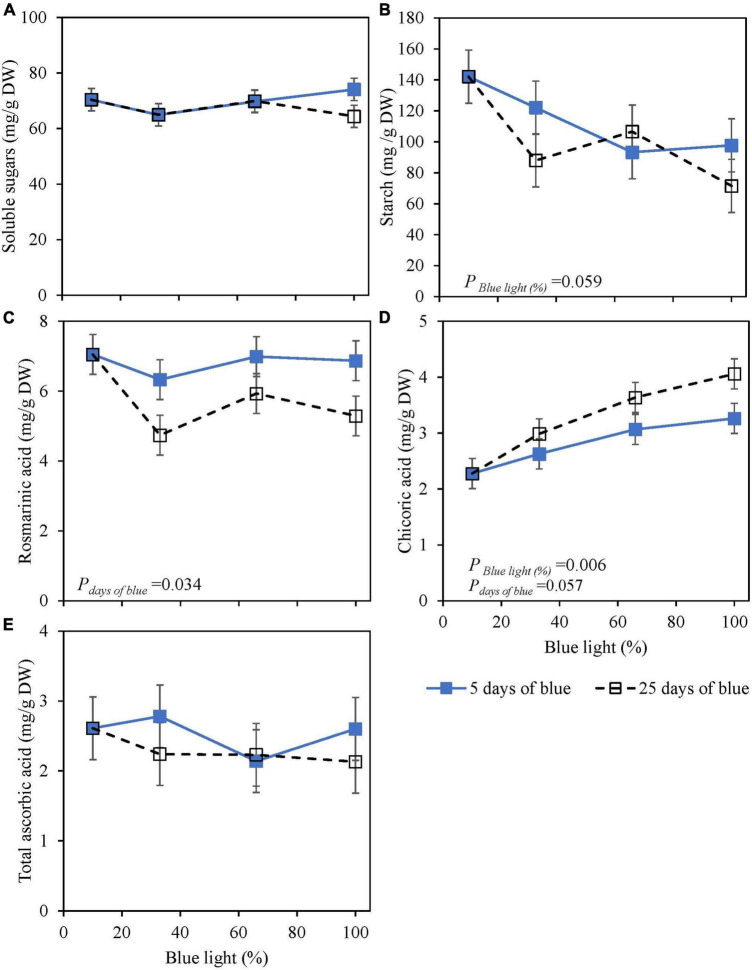
Metabolite levels at harvest in basil cv. Dolly exposed either continuously (25 days) (open symbols) or for 5 days of End-Of-Production (EOP) (closed symbols) to different percentages of blue light. Plants were harvested after 40 days of cultivation. After 15 days, plants were transplanted. After the transplant, the continuously treated plants grew under different percentages of blue light until harvest [photon flux density (PPFD) of 300 μmol m^–2^ s^–1^]. The EOP plants were grown under red-white (PPFD of 300 μmol m^–2^ s^–1^, 9% blue) light and later exposed to different percentages of blue light (PPFD of 300 μmol m^–2^ s^–1^) for the last 5 days before harvest as EOP treatments. **(A)** Soluble sugars (sum of glucose, fructose, and sucrose), **(B)** starch, **(C)** rosmarinic acid, **(D)** chicoric acid, **(E)** total ascorbic acid. All values are expressed per gram dry weight in the leaves. The data are means of two blocks (*n* = 2) (i.e., per block four replicate plants). Standard errors of means are shown as error bars. Significance of the main effects percentage of blue and days of blue (α = 10%) are depicted (Experiment 1).

Soluble sugars (glucose, fructose, and sucrose) at harvest were not affected by the percentage of blue light during cultivation (25 days) or 5 days of EOP. Starch content was reduced by 30–50% with an increasing percentage of blue light for both EOP and continuously treated plants (Figures 1A,B). Rosmarinic acid at harvest was little affected by the percentage of blue. However, continuous blue (33, 65, and 100% blue) treatments resulted in a 15–25% decrease compared to the shorter duration of EOP blue treatments (Figure 1C). Chicoric acid levels increased with an increasing percentage of blue whether it was provided continuously throughout cultivation or EOP, but the increase was stronger when applied throughout cultivation (+85%) compared to EOP (+45%) (Figure 1D). TAsA did not respond to either percentage of blue or duration of blue light (Figure 1E).

The changes in metabolites from the EOP treated plants were measured during postharvest storage. During storage at both 4 and 12°C, sugars increased whereas starch decreased over time (Supplementary Figure 1). However, the patterns of the time courses were not affected by the EOP blue light treatments. During postharvest storage at 4°C, rosmarinic acid, chicoric acid, and AAsA showed a steep decrease over time (Supplementary Figure 2). At 12°C, an initial increase was observed in chicoric and rosmarinic acids for all treatments, later followed by a decrease. TAsA at 12°C showed a similar pattern as at 4°C (Supplementary Figure 2).

### Metabolite Content in Response to the Percentage of Blue Light at Different PPFD

We investigated the interactive effect between the percentage of blue light (low; 9% or high; 90%) and PPFD (low; 100 μmol m^–2^ s^–1^, or high; 300 μmol m^–2^ s^–1^) applied as EOP treatment the last 5 days before harvest on metabolites in green cv. Dolly and purple cv. Rosie (Experiment 2). The high PPFD and the low blue were the same as in Experiment 1.

**FIGURE 2:**
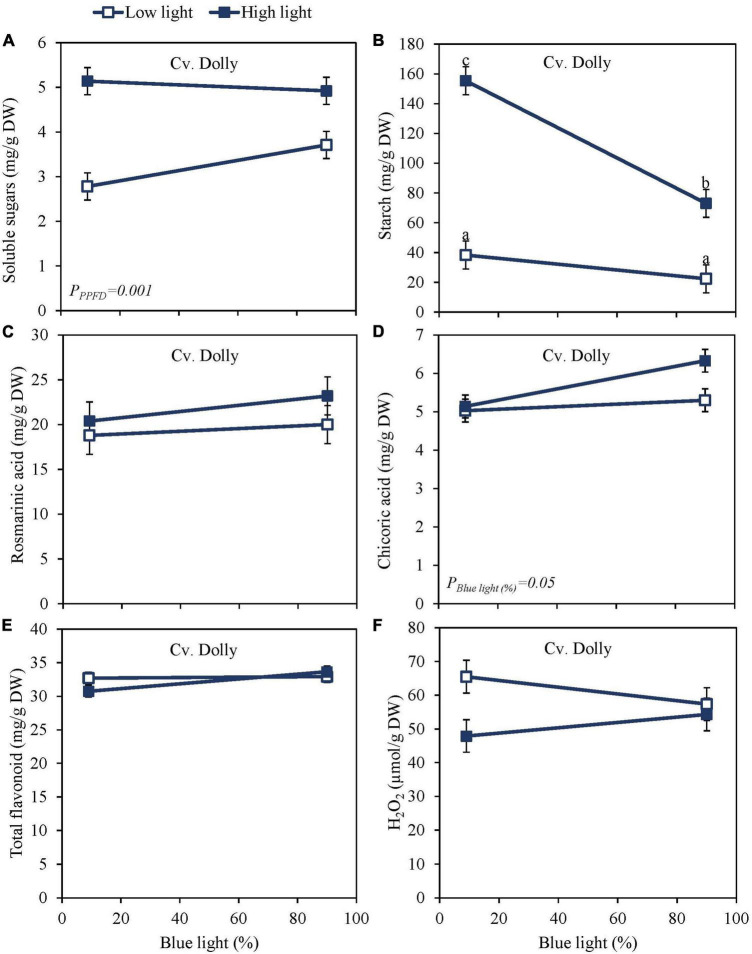
Metabolite levels at harvest in basil, the green cv. Dolly were exposed to either 9% or 90% blue light at low PPFD (100 μmol m^–2^ s^–1^) (open symbols) or high PPFD (300 μmol m^–2^ s^–1^) (closed symbols) applied the last 5 days before harvest as EOP treatments. Before EOP treatments plants were grown under red-white light (PPFD of 200 μmol m^–2^ s^–1^, 9% blue) for 30 days. **(A)** Soluble sugars (sum of glucose, fructose, and sucrose), **(B)** starch, **(C)** rosmarinic acid, **(D)** chicoric acid, **(E)** total flavonoids, **(F)** hydrogen peroxide (H_2_O_2_). All values are expressed per gram dry weight in the leaves. The data are means of three blocks (*n* = 3) (i.e., per block four replicate plants). Standard errors of means are shown as error bars. Significance of the main effects percentage of blue light and PPFD (α = 5%) are shown. Letters indicate and interactive effect between the two main effects (percentage of blue light and PPFD), (Experiment 2).

**FIGURE 3:**
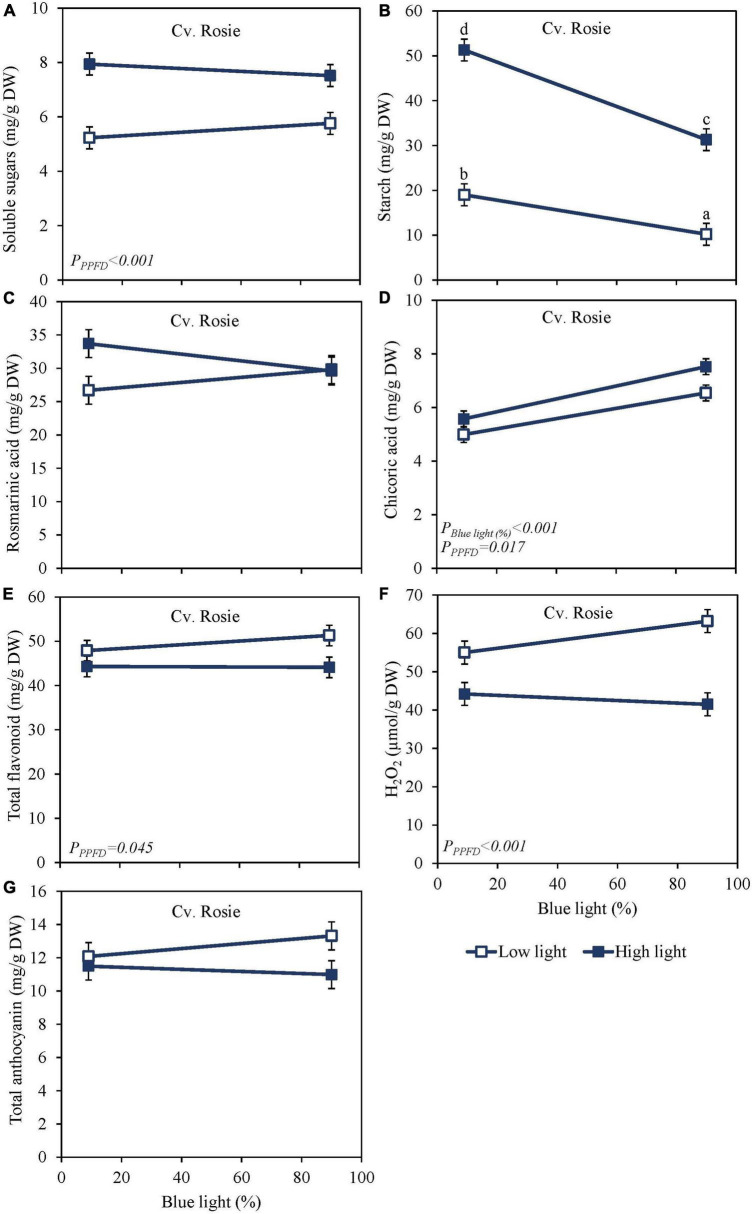
Metabolite levels at harvest in basil the purple cv. Rosie were exposed to either 9% or 90% blue light at low PPFD (100 μmol m^–2^ s^–1^) (open symbols) or high PPFD (300 μmol m^–2^ s^–1^) (closed symbols) applied the last 5 days before harvest as EOP treatments. Before EOP treatments plants were grown under red-white light (PPFD of 200 μmol m^–2^ s^–1^, 9% blue) for 30 days. **(A)** Soluble sugars (sum of glucose, fructose, and sucrose), **(B)** starch, **(C)** rosmarinic acid, **(D)** chicoric acid, **(E)** total flavonoids, **(F)** H_2_O_2_, **(G)** total anthocyanins. All values are expressed per gram dry weight in the leaves. The data are means of four blocks (*n* = 4) (i.e., per block four replicate plants). Standard errors of means are shown as error bars. Significance of the main effects percentage of blue light and PPFD (α = 5%) are shown. Letters indicate and interactive effect between the two main effects (percentage of blue light and PPFD), (Experiment 2).

High PPFD as EOP treatment increased soluble sugars and starch content at harvest in both the green cv. Dolly (Figures 2A,B) and the purple cv. Rosie (Figures 3A,B). Furthermore, for both cultivars, starch content was significantly higher in the high PPFD treatment combined with low blue compared to the high PPFD with high blue. A higher percentage of blue light increased the content of chicoric acid in both cultivars (Figures 2D, 3D), while high PPFD also increased the content of chicoric acid in purple cv. Rosie. Rosmarinic acid was neither affected by PPFD nor by the percentage of blue light (Figures 2C, 3C). Similarly, the total anthocyanin content in the purple cv. Rosie was not significantly affected by either PPFD or the percentage of blue light (Figure 3G). The green cv. Dolly did not contain anthocyanins. In contrast, total flavonoid content and the level of H_2_O_2_ were lower at high PPFD than at low PPFD in purple cv. Rosie (Figures 3E,F). However, for neither the green nor the purple cultivar did percentage of blue light and PPFD have an interactive effect on the content of metabolites.

Sugar levels were slightly higher and starch levels were considerably lower in purple cv. Rosie compared to green cv. Dolly (Figures 2A, 3A). Rosmarinic acid, chicoric acid, and flavonoids were higher in purple cv. Rosie compared to green cv. Dolly. The purple cv. Rosie contained anthocyanin, which was absent (below the detection level) in the green cv. Dolly. Together it implies the purple cv. Rosie had a higher level of antioxidants (secondary metabolites) but a considerably lower level of carbohydrate reserves at harvest.

From Experiment 1 with the green cv. Dolly, it was clear that the pre-harvest blue light had little or no effects on the metabolite changes during the postharvest phase. Therefore, in Experiment 2 the green cv. Dolly was sampled only on days 3 and 6 during storage at 4 and 12°C. The purple cultivar Rosie was sampled in addition also at days 9 and 12. During storage soluble sugars increased at both 4 and 12°C for the green cv. Dolly (Supplementary Figures 3A,B) whereas for the purple cv. Rosie a slight decrease was observed at 12°C (Supplementary Figure 4B). The levels of sugars in the postharvest phase were generally higher in the samples derived from plants from high PPFD EOP treatments while the percentage of blue light did not have an effect on the postharvest content. During postharvest storage at both 4 and 12°C, the starch content remained the highest from high PPFD and low blue EOP treatments in both cvs Dolly and Rosie (Supplementary Figures 3C,D, 4C,D). The starch reserves, especially in the samples from low PPFD and high PPFD/high percentage of blue light treatments were depleted by days 3–6 in the purple cv. Rosie. At that time, depletion was not complete in the green cv. Dolly had higher starch levels at harvest. The starch breakdown was generally faster at 12 than at 4°C.

**FIGURE 4:**
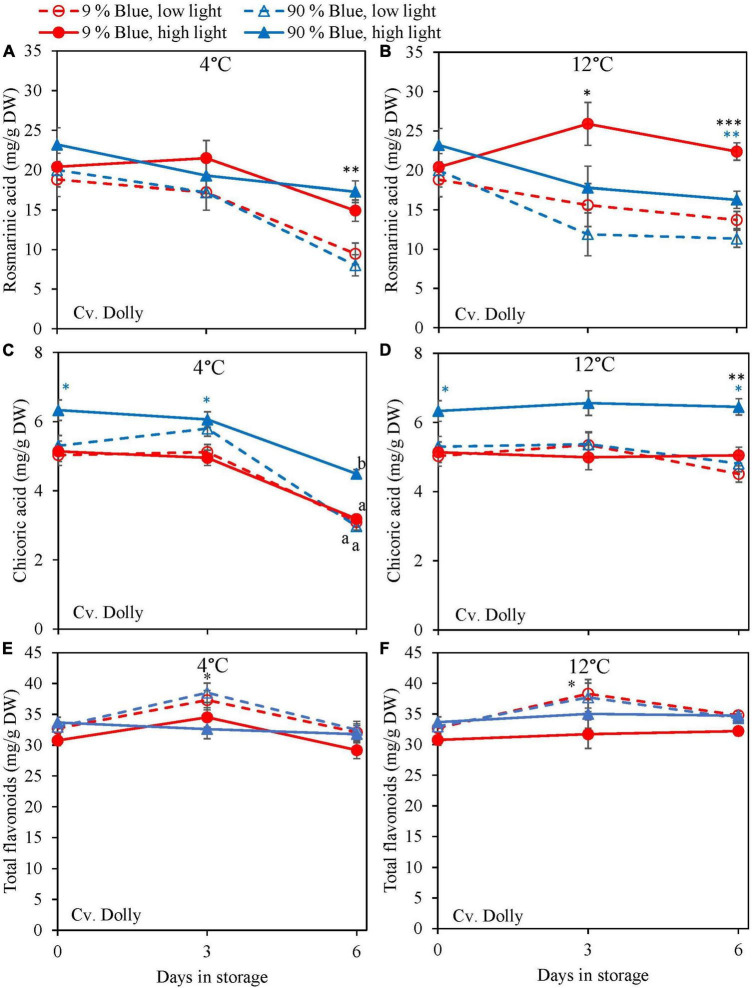
Changes in metabolite levels during postharvest storage at 4 **(A,C,E)** and 12°C **(B,D,F)** in basil in the green cv. Dolly were exposed to EOP treatments. Plants were grown under red-white light (PPFD of 200 μmol m^–2^ s^–1^, 9% blue) for 30 days. The last 5 days before harvest plants were exposed to different EOP blue light ratios 9 or 90% at low PPFD (100 μmol m^–2^ s^–1^) (open symbols) or high PPFD (300 μmol m^–2^ s^–1^) (closed symbols). **(A,B)** Change in rosmarinic acid, **(C,D)** change in chicoric acid, **(E,F)** change in total flavonoid content. All values are expressed per gram dry weight in the leaves. The data are means of three blocks (*n* = 3) (i.e., per block four replicate plants). Standard errors of means are shown as error bars. If no interaction was found but only the main effects were significant the indicated with *p*-values; *p < 0.05, ^**^p < 0.01, ^***^p < 0.001 are depicted with either a blue (percentage of blue light) or black asterisk (PPFD). Letters indicate and interactive effect between the two main effects (percentage of blue light and PPFD), (Experiment 2).

**FIGURE 5:**
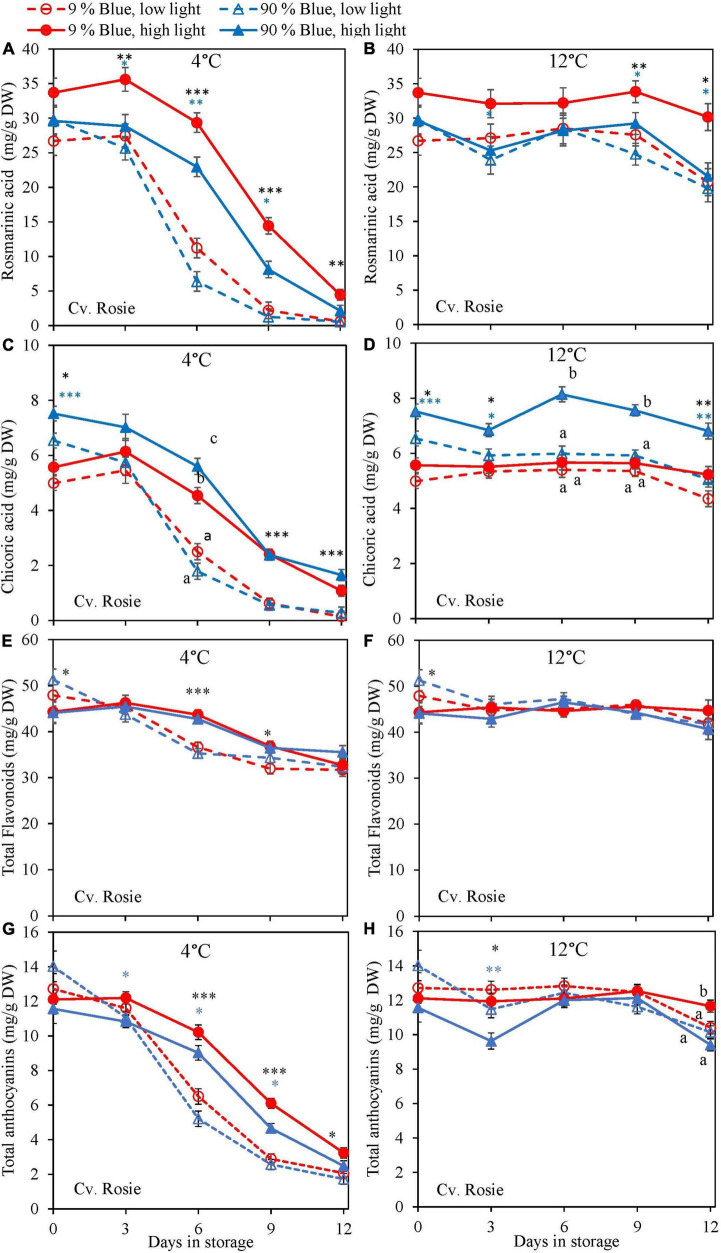
Changes in metabolite levels during postharvest storage at 4 **(A,C,E,G)** and 12°C **(B,D,F,H)** in basil in the purple cv. Rosie were exposed to EOP treatments. Plants were grown under red-white light (PPFD of 200 μmol m^–2^ s^–1^, 9% blue) for 30 days. The last 5 days before harvest plants were exposed to different EOP blue light ratios 9 or 90% at low PPFD (100 μmol m^–2^ s^–1^) (open symbols) or high PPFD (300 μmol m^–2^ s^–1^) (closed symbols). **(A,B)** Change in rosmarinic acid, **(C,D)** change in chicoric acid, **(E,F)** change in total flavonoid content, **(G,H)** change in total anthocyanin content. All values are expressed per gram dry weight in the leaves. The data are means of four blocks (*n* = 4) (i.e., per block four replicate plants). Standard errors of means are shown as error bars. If no interaction was found but only the main effects were significant the indicated with *p*-values; *p < 0.05, ^**^p < 0.01, ^***^p < 0.001 are depicted with either a blue (percentage of blue light) or black asterisk (PPFD). Letters indicate and interactive effect between the two main effects (percentage of blue light and PPFD), (Experiment 2).

Overall, metabolites were unchanged at 12°C storage for both cultivars (Figures 4, 5) but in the purple cv. Rosie a pronounced decrease in the metabolite levels was observed at 4°C. During the 6 days of storage, there was no clear effect of the EOP light treatments on the changes in metabolite levels in the green cv. Dolly. For the purple cv. Rosie, high PPFD during cultivation resulted in a slower decrease of metabolites (rosmarinic acid, chicoric acid, total flavonoid content, and total anthocyanin content) during dark storage at 4°C (Figures 5A,C,E,G). In addition, a low percentage of blue light resulted in a slower decrease of rosmarinic acid and total anthocyanin content compared to a high percentage of blue light in the purple cv. Rosie at 4°C (Figures 5A,G). The reverse effect was seen for chicoric acid, where a high percentage of blue light resulted in the slowest decrease for both cvs (Figures 4C, 5C).

### Chilling Tolerance in Response to the Percentage of Blue Light and PPFD

**FIGURE 6:**
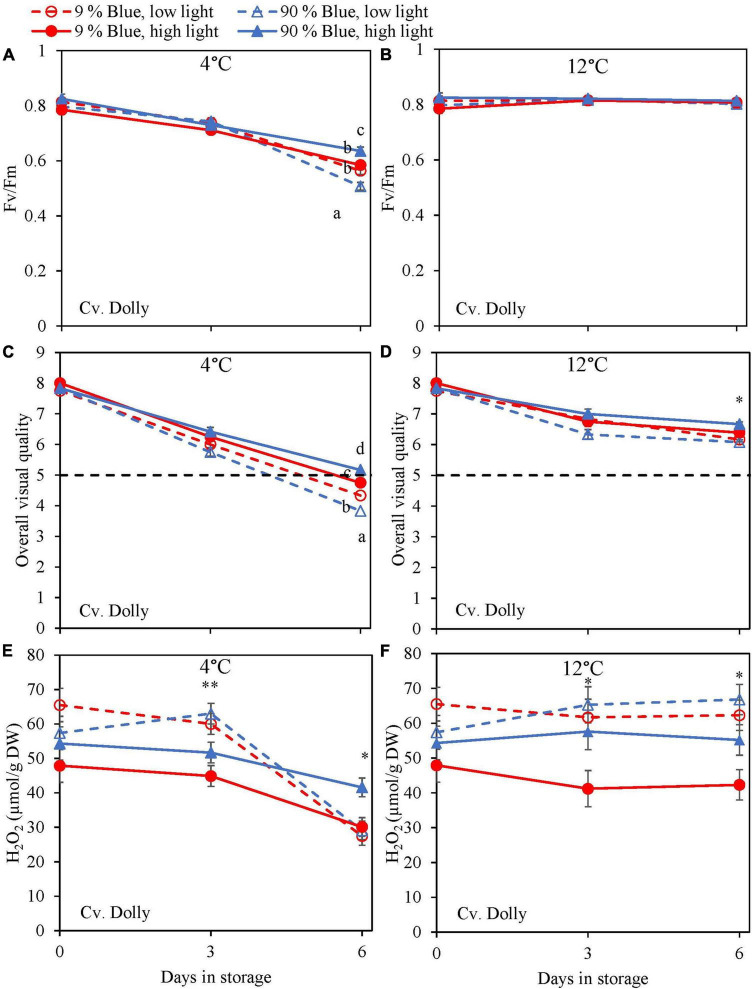
Changes in chilling injury parameters during postharvest storage at 4 **(A,C,E)** and 12°C **(B,D,F)** in basil in the green cv. Dolly were exposed to EOP treatments. Plants were grown under red-white light (PPFD of 200 μmol m^–2^ s^–1^, 9% blue) for 30 days. The last 5 days before harvest plants were exposed to different EOP blue light ratios 9 or 90% at low light (100 μmol m^–2^ s^–1^) (open symbols) or high light (300 μmol m^–2^ s^–1^) (closed symbols). **(A,B)** Change in maximum quantum yield of PSII of dark-adapted leaves (F_*v*_/F_*m*_), **(C,D)** change Overall Visual Quality (OVQ), **(E,F)** change in hydrogen peroxide (H_2_O_2_) content. All metabolite values are expressed per gram dry weight in leaves. The data are means of three blocks (*n* = 3) (i.e., per block four replicate plants). Standard errors of means are shown as error bars. If no interaction was found but only the main effects were significant the indicated with *p*-values; *p < 0.05, **p < 0.01, are depicted with either a blue (percentage of blue light) or black asterisk (PPFD). Letters indicate and interactive effect between the two main effects (percentage of blue light and PPFD), (Experiment 2).

**FIGURE 7:**
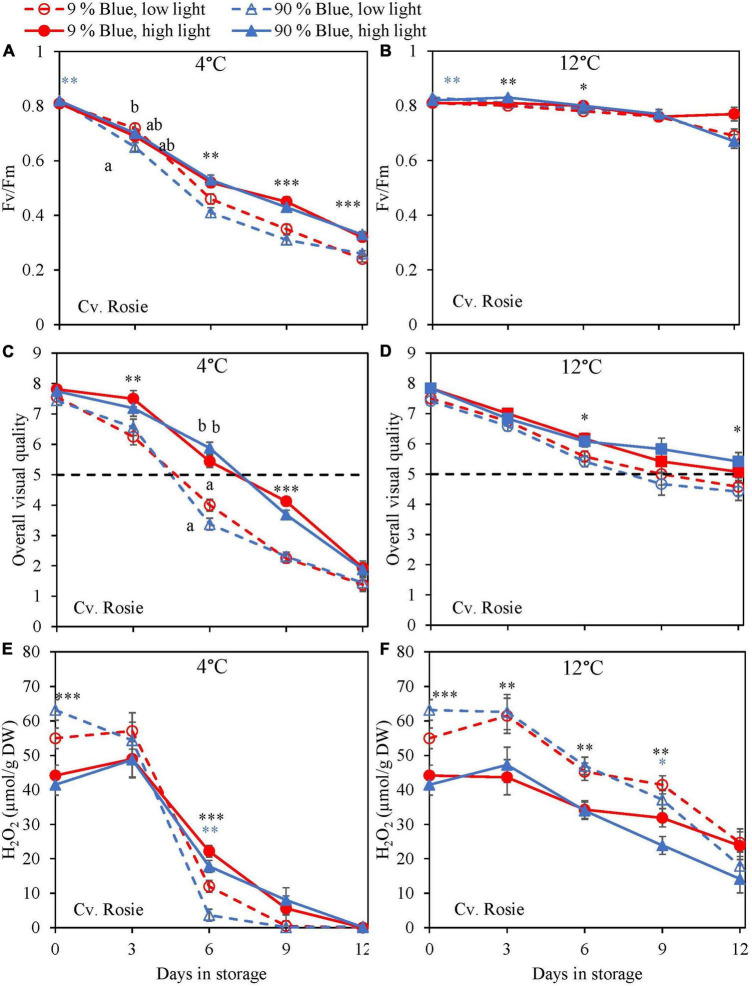
Changes in chilling injury parameters during postharvest storage at 4 **(A,C,E)** and 12°C **(B,D,F)** in basil in the purple cv. Rosie was exposed to EOP treatments. Plants were grown under red-white light (PPFD of 200 μmol m^–2^ s^–1^, 9% blue) for 30 days. The last 5 days before harvest plants were exposed to different EOP blue light ratios 9 or 90% at low light (100 μmol m^–2^ s^–1^) (open symbols) or high light (300 μmol m^–2^ s^–1^) (closed symbols). **(A,B)** Change in Maximum quantum yield of PSII of dark-adapted leaves (F_*v*_/F_*m*_), **(C,D)** change in OVQ, **(E,F)** change in H_2_O_2_ content. All metabolite values are expressed per gram dry weight in leaves. The data are means of four blocks (*n* = 4) (i.e., per block four replicate plants). Standard errors of means are shown as error bars. If no interaction was found but only the main effects were significant the indicated with *p*-values; *p < 0.05, ^**^p < 0.01, ^***^p < 0.001 are depicted with either a blue (percentage of blue light) or black asterisk (PPFD). Letters indicate and interactive effect between the two main effects (percentage of blue light and PPFD), (Experiment 2).

During the storage, F_*v*_/F_*m*_ was measured as a marker for chilling injury. During dark storage at 12°C, there was little change over time and no effect of PPFD or the percentage of blue light during cultivation (Figures 6B, 7B). For both cultivars, high PPFD during cultivation resulted in less chilling injury during 4°C storage (i.e., a slower decrease of F_*v*_/F_*m*_ and a longer shelf life) (Figures 6A, 7A). The high percentage of blue light showed a minor effect on the chilling injury for both cultivars. OVQ values were in line with F_*v*_/F_*m*_ values. At 1°C, OVQ values slowly decreased and there were no clear effects of EOP PPFD or percentage of blue light (Figures 6D, 7D). At 4°C a high PPFD had a positive effect on OVQ values for both cultivars (Figures 6C, 7C) whereas the effect of percentage of blue light was limited.

During chilling injury, the content of H_2_O_2_ (i.e., a ROS) may increase due to chilling stress. At 12°C, the H_2_O_2_ content remained unchanged in the green cv. Dolly but decreased in the purple cv. Rosie (Figures 6F, 7F). H_2_O_2_ levels in samples from low PPFD EOP treatments were generally higher than in samples from high PPFD in both cvs. There was no effect on the percentage of blue light. At 4°C, H_2_O_2_ levels in both cvs rapidly decreased, the decrease seemed to be more pronounced in samples from low PPFD EOP treatments; there was no effect of percentage of blue light on the observed patterns (Figures 6E, 7E).

## Discussion

### Blue Light Did Not Stimulate the Biosynthesis of Secondary Metabolites From the Phenylpropanoid Pathway

Blue light has been reported in numerous studies to increase the content of antioxidants in several plant species such as strawberry, green and red lettuce, green and red pak choi, and Arabidopsis (Chen et al., 2006; Samuoliene et al., 2013; Ouzounis et al., 2015; Zhang et al., 2018; Zheng et al., 2018). Thus, we hypothesized that blue light would increase the content of antioxidants at harvest in green and purple basil. Our findings indicate that a high percentage of blue light (up to 100%) during the whole cultivation period applied as a 5-day EOP treatment did not increase the content of rosmarinic acid in basil leaves (Figures 1C, 2C, 3C). Similar to Taulavuori et al. (2016), we found that a continuous application of a high percentage of blue light even negatively affected the content of rosmarinic acid (Figure 1C). Spectra that earlier have been reported to result in a high content of rosmarinic acid included red with supplementary far-red (Schwend et al., 2016), red light (Shiga et al., 2009), and blue light under greenhouse conditions. However, these studies did not consistently compare how the different percentages of the spectra affect the biosynthesis of rosmarinic acid (i.e., percentage of blue vs. green, red, and far-red). Chicoric acid generally constitutes a minor amount of the total level of phenolic compounds in the basil cultivars under study (Figures 1D, 2D, 3D). Although chicoric acid has been found to be a stronger antioxidant than rosmarinic acid (Dalby-Brown et al., 2005), it is unknown to what extent chicoric acid contributes to the overall scavenging activity of basil antioxidants. Similar to our results, Taulavuori et al. (2016) found blue light to have a positive effect on the content of chicoric acid. We found different absolute levels of especially rosmarinic acid content in the two experiments. However, the response to blue light remained the same between the two experiments (i.e., no effect of blue light). The main difference between the experiments was the initial PPFD; Experiment 1 had a PPFD of 300 μmol m^–2^ s^–1^ and Experiment 2 a PPFD of 200 μmol m^–2^ s^–1^. Similar to the high content of rosmarinic acid in Experiment 2 our previous results in basil also showed increased PPFD as EOP treatment to increase rosmarinic acid (Larsen et al., 2022). Furthermore, the duration of Experiment 1 was 40 days while Experiment 2 was 35. The younger leaves in Experiment 2 may have additionally contributed to a higher content of rosmarinic acid at harvest.

Blue light is absorbed by photoreceptors such as cryptochromes (cry1 and cry2) which mediates plant responses. The genes involved in the biosynthesis of flavonoids and anthocyanins through the phenylpropanoid pathway are induced through cry1 (Jenkins et al., 2001). The starting point for the flavonoid biosynthesis branch is chalcone synthase (CHS). The expression level of *CHS* has been found to increase by blue light in Arabidopsis cells (Christie and Jenkins, 1996) and increase anthocyanin and flavonoid content in several species. In pepper leaves, increasing blue light to 75% increased the content of anthocyanins but not flavonoids (Hoffmann et al., 2016). However, in our study total anthocyanin content did not increase with an increased percentage of blue light (Figure 3G), and neither did total flavonoid content in the green cultivar (Figure 2E) nor the purple cultivar (Figure 3E). Similarly, Dou et al. (2019) found that increasing the blue light from 16 to 24% did not increase the anthocyanin content in green or purple basil while they did find a positive effect on flavonoids in green basil. Findings by Pennisi et al. (2019) indicated an optimum at 23% blue light resulting in the highest total flavonoids content while 58% and 19% blue light resulted in the lowest total flavonoid content in green basil. In contrast, Piovene et al. (2015) found that a range of blue light from 10 to 40% did not increase the total flavonoid content in basil. In red lettuce, 47% blue light gave the highest content of flavonoids whereas 59% blue light yielded the highest content in green lettuce (Son and Oh, 2013). Although it is generally assumed that blue light will increase particularly the flavonoid and anthocyanin content there is no general consistency in the results; this makes it hard to predict which percentage of blue light may be required to increase the flavonoid and anthocyanin content if any. However, it should be noted that changes in the percentage of blue light also cause changes in the contribution of other wavelengths, that may contribute to the measured effects. Based on our results it seems that a low percentage of blue light (9%) in the spectrum is already sufficient for maximal biosynthesis of phenolic compounds (phenolic acids, flavonoids, and anthocyanins) in green and purple basil.

### The Content of Secondary Metabolites in Response to the Percentage of Blue Light Did Not Depend on PPFD

We hypothesized that the response to blue light might be reduced by high PPFD (300 μmol m^–2^s^–1^). The accumulation of antioxidants in response to blue light may be more pronounced at a lower PPFD compared to a high PPFD as a high PPFD regardless of the spectrum leads to an increase in antioxidants (Larsen et al., 2022). In cannabis, the percentage of blue light at a PPFD of 750 and 900 μmol m^–2^s^–1^ did not affect secondary metabolites which were assumed to be attributed to saturated photoreceptors (i.e., less sensitivity to spectral effects) (Westmoreland et al., 2021). We tested if the effect of varying percentages of blue light was different when applied on a low or high PPFD background. We did not find such an effect on the metabolite content in either green or purple basil. This is in contrast to findings by Zheng et al. (2018) in green and red-leaved pak choi where the percentage of blue light and PPFD had an interactive effect on vitamin C, carotenoids, and total phenolic content.

At harvest, the dominant effect of light on metabolites came from the PPFD (Figures 2, 3) which is in line with the results of Larsen et al. (2022) where increased PPFD increased both primary and secondary metabolites in basil. In red lettuce, anthocyanin and phenolic acids were not affected by PPFD whereas flavonoids increased (Becker et al., 2013). This is in accordance with our results where PPFD had no effect on anthocyanin content but a small increasing effect on flavonoid content at harvest (Figure 3E). In studies where anthocyanins have been reported to increase with an increase in PPFD, the much bigger difference between light intensities was applied; from 100 to 550–650 μmol m^–2^ s^–1^ (Page et al., 2012) from 50–350 to 750 μmol m^–2^s^–1^ (Albert et al., 2009). To get an overview of the whole plant, we sampled all fully developed leaves at harvest except the oldest leaf pair. Young leaves contain higher amounts of anthocyanins than older leaves and by sampling young leaves along with more mature leaves, we could potentially have had a dilution effect (Chalker-Scott, 1999). In addition to antioxidants, we found that starch content was significantly decreased at a high PPFD and a high percentage of blue light in both green and purple basil (Figures 1B, 2B, 3B), this could negatively affect shelf life as starch is used for respiration (Enninghorst and Lippert, 2003). Although, we found that starch was decreased in both experiments the dry mass of the leaves only decreased with the percentage of blue light when continuously grown under a high PPFD (300 μmol m^–2^ s^–1^) (Larsen et al., 2020).

### High PPFD Resulted in a Slower Breakdown of Antioxidants and Improved Chilling Tolerance Postharvest

In lettuce (Min et al., 2021) and basil (Larsen et al., 2022), it was found that the stimulating effect of high PPFD on metabolites observed at harvest was maintained during postharvest storage. In the present experiments, levels at harvest were little affected by PPFD, but during postharvest storage at 4°C antioxidants (rosmarinic acid, chicoric acid, total anthocyanin content, and total flavonoid content) in the purple cv. Rosie from high PPFD EOP light treatments showed a slower breakdown than in samples from low PPFD treatments (Figure 5). The effect of PPFD on the rate of the postharvest breakdown of antioxidants was less pronounced in the green cv. Dolly (Figure 4). The slower breakdown of antioxidants during storage at 4°C coincided with a slower decrease in F_*v*_/F_*m*_ and Overall visual quality (OVQ) (Figures 6A,C, 7A,C). This indicates that the plants from high PPFD were more tolerant to the cold. Although antioxidants may scavenge ROS and in turn result in maintaining high F_*v*_/F_*m*_ values we believe that another mechanism could also be in play here. In the purple cv. Rosie, high light did increase the soluble sugar and starch content (Supplementary Figure 4) which can protect against chilling stress (Santarius, 1973). Plants with an increased chilling tolerance may have had higher levels of hormones such as ABA and JA (Lado et al., 2016; Wang et al., 2016). However, this has yet to be studied in basil.

During excessive stress, H_2_O_2_ is not only formed in the mitochondria and chloroplasts but also in the peroxisomes resulting in lipid peroxidation (Corpas et al., 2001). Exposure of basil to chilling temperatures was expected to increase the content of H_2_O_2_. However, during storage at 4°C, we observed a strong decrease of H_2_O_2_ in both cultivars (Figures 6E, 7E). In contrast, the level of H_2_O_2_ at 12°C either remained constant in the green cv. Dolly (Figure 6F) or showed a slow decrease in the purple cv. Rosie (Figure 7F). The low H_2_O_2_ concentration at 4°C may be a result of scavenging by anthocyanins, flavonoids, rosmarinic acid, and chicoric acid, which all showed comparable decreasing trends at 4°C (Figures 2, 3). Enzymatic antioxidants might also have been active in scavenging as the content is known to increase when plants are grown under high PPFD (Ali et al., 2005; Zhou et al., 2012).

## Conclusion

Contrary to our hypothesis and general expectations, a high percentage of blue light applied either continuously throughout the growth or as EOP treatment did not increase antioxidants such as rosmarinic acid, total anthocyanin content, or total flavonoid content at harvest. The only antioxidant that was increased by a percentage of blue light was chicoric acid, which is only a minor part of the total antioxidant content. The absence of effects of blue light was observed both in green and purple basil cultivars, and the absence was also observed whether PPFD was high or low. Chilling tolerance is supposed to be related to the scavenging activity of antioxidants. The lack of effect of the percentage of blue light on antioxidant levels is in line with the absence of a percentage of blue light effects on chilling tolerance. Although a high percentage of blue light did not improve postharvest chilling tolerance in green or purple basil, high PPFD EOP treatments did. High PPFD as EOP treatment particularly improved chilling tolerance in the purple cultivar, reflected in a slower decrease in antioxidants than in samples from in low PPFD treatments. This may not be related to the levels of antioxidants but to the higher carbohydrate levels (soluble sugars and starch) in leaves from high PPFD grown plants.

## Data Availability Statement

The raw data supporting the conclusions of this article will be made available by the authors, without undue reservation.

## Author Contributions

DL, EW, CN, JV, and LM conceptualized the research plan. DL, EW, and LM designed the experiments. DL and HL established the methodology. DL and SS carried out the experiments. DL analyzed the data and wrote the manuscript. EW and LM provided critical feedback on the manuscript and supervised the research. CN, HL, JV, and SS provided critical comments on the overall structure of the manuscript. All authors reviewed and approved the final manuscript.

## Conflict of Interest

CN was employed by Signify Research Laboratories Ltd. The remaining authors declare that the research was conducted in the absence of any commercial or financial relationships that could be construed as a potential conflict of interest.

## Publisher’s Note

All claims expressed in this article are solely those of the authors and do not necessarily represent those of their affiliated organizations, or those of the publisher, the editors and the reviewers. Any product that may be evaluated in this article, or claim that may be made by its manufacturer, is not guaranteed or endorsed by the publisher.
