# Supplementary material for: Lack of Blue Light Regulation of Antioxidants and Chilling Tolerance in Basil
Source: Front Plant Sci. 2022 Apr 7;13:852654. doi: 10.3389/fpls.2022.852654 (PMC9021895; doi:10.3389/fpls.2022.852654)
Supplement: Supplementary file 1 [file Data_Sheet_1.docx]

Supplementary Material

**Table S1**. Description of postharvest appearance of leaves and the corresponding overall visual quality score. Scores are ranging from 8-1, with 8 being the highest. The consumer acceptance limit is set at the score 5.

| OVQ score | Description of postharvest appearance |
| --- | --- |
| 8 | Leaves are crisp, do not show dark spots + dark green color/dark purple color, initial leaf shininess, no wilting, curved leaf shape. |
| 7 | Slight loss of initial crispness, do not show dark spots + color change, slight loss of initial leaf shininess, no wilting and show slight loss of initial curved leaf shape. |
| 6 | Leaves are moderately crisp, show none/very limited dark spot presence + color change, moderate leaf shininess, no wilting, slight loss of curved leaf shape. |
| 5 | Indicates end of shelf-life. Leaves are moderately crisp, show some dark spot presence + color change, moderately shiny, some/very limited wilting signs, moderately curved leaf shape. |
| 4 | Overall loss of crispness, obvious and widespread overall dark spot (< 50% total leaf area) presence + color change, overall loss of shininess, moderate wilting, overall loss of curved leaf shape. |
| 3 | Leaves are not crisp, obvious and widespread (>50% total leaf area) overall dark spot presence + color change, show no leaf shininess, moderate wilting, no curved leaf shape. |
| 2 | Leaves are not crisp, obvious and widespread (>50% total leaf area) overall dark spot presence + color change, show no leaf shininess, completely wilted, no curved leaf shape, ‘wet leaves’ (ion leakage). |
| 1 | Leaves are not crisp, obvious and widespread (>50% total leaf area) overall dark spot presence + color change, show no leaf shininess, completely wilted , no curved leaf shape, completely ‘wet leaves’ (ion leakage) |


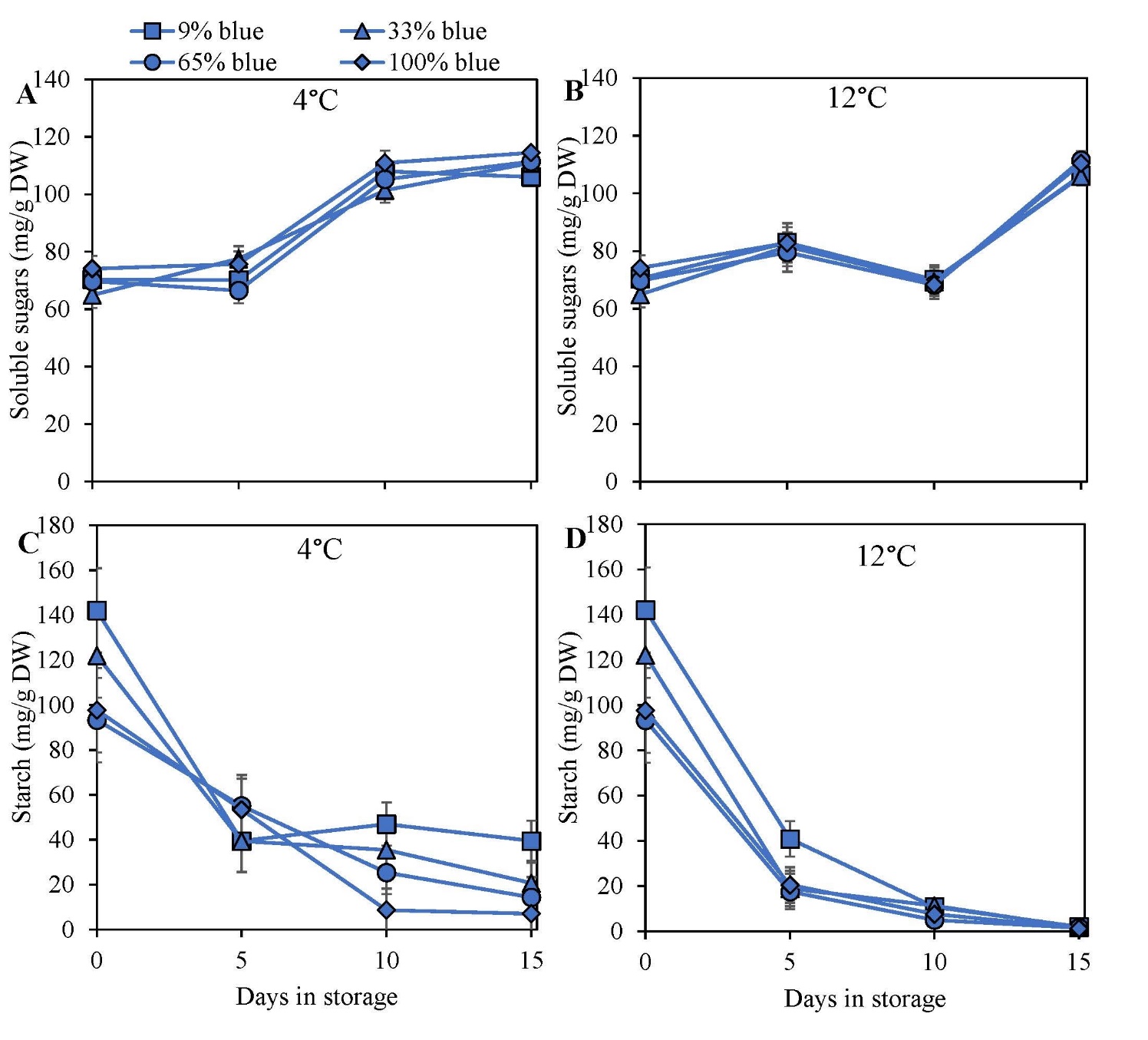
**Figure S1.** Changes in metabolite levels during postharvest storage at 4 (panels A,C,) and 12° C (panels B,D,) in basil cv Dolly. Plants were previously treated with different EOP to different blue ratios (Exp 1). A and B. change in soluble sugars (sum of glucose, fructose and sucrose), C and D, change in starch. Data are means of 2 blocks (n=2) with 4 replicate plants per block. Error bars represent standard errors of means, when larger than symbols. Letters indicate significant differences (α=10%) at individual time points.


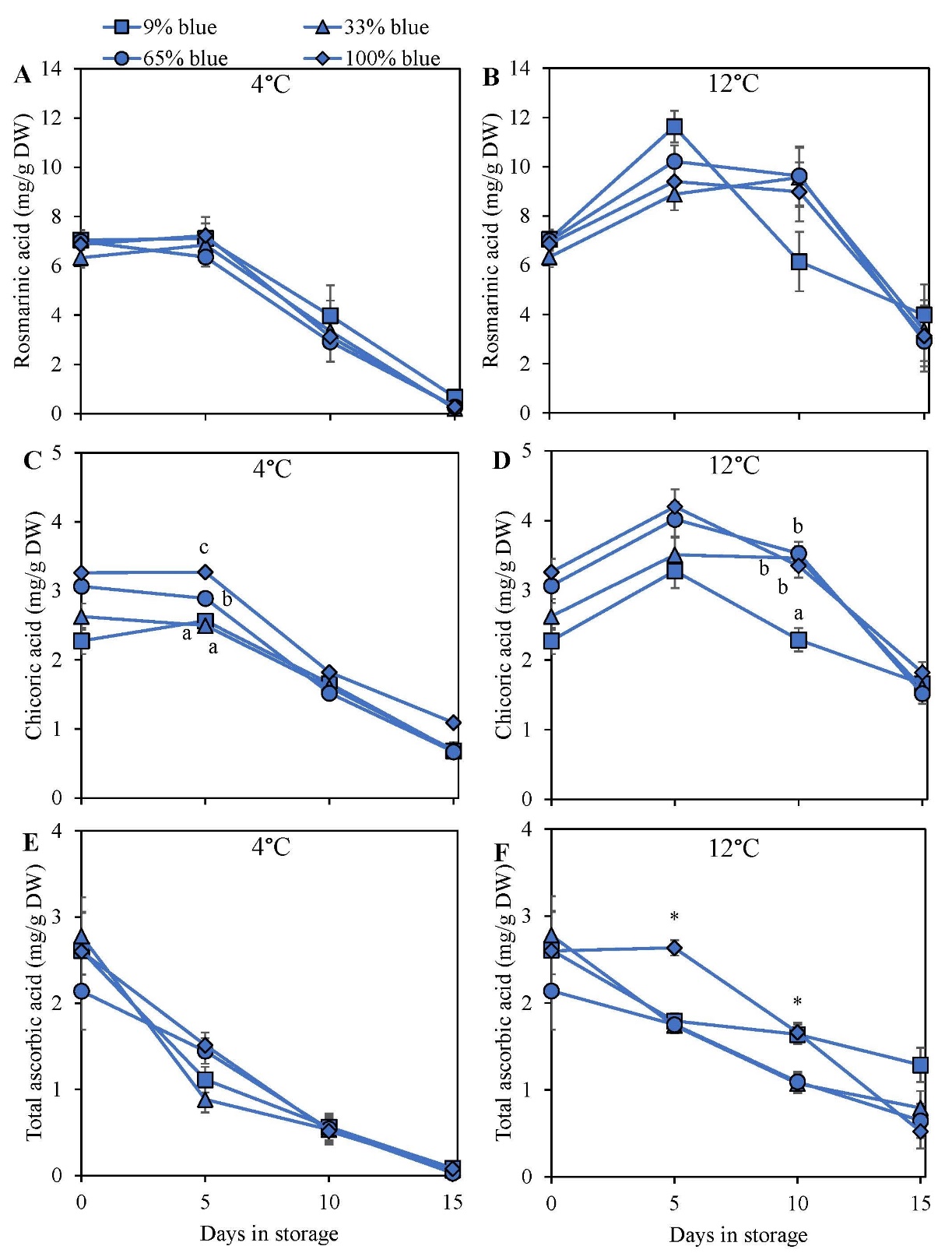


**Figure S2.** Changes in metabolite levels during postharvest storage at 4 (panels A,C,E) and 12° C (panels B,D,F) in basil cv Dolly exposed to End-Of-Production (EOP) treatments. Plants were harvested after 40 days of cultivation. Plants were previously grown under red-white (PPFD of 300 µmol m^-2^ s^-1^, 9% blue) light and later exposed to different %blue light (PPFD of 300 µmol m^-2^ s^-1^) the last five days before harvest as EOP treatment (PPFD of 300 µmol m^-2^ s^-1^). A and B. change in rosmarinic acid, C and D. change in chicoric acid, E and F. change in total ascorbic acid. All values are expressed per gram dry weight in the leaves. The data are means of two blocks (n=2) (i.e. per block four replicate plants). Standard errors of means are shown as error bars. Letters indicate significant differences (α=10%) at individual time points, (Exp. 1).


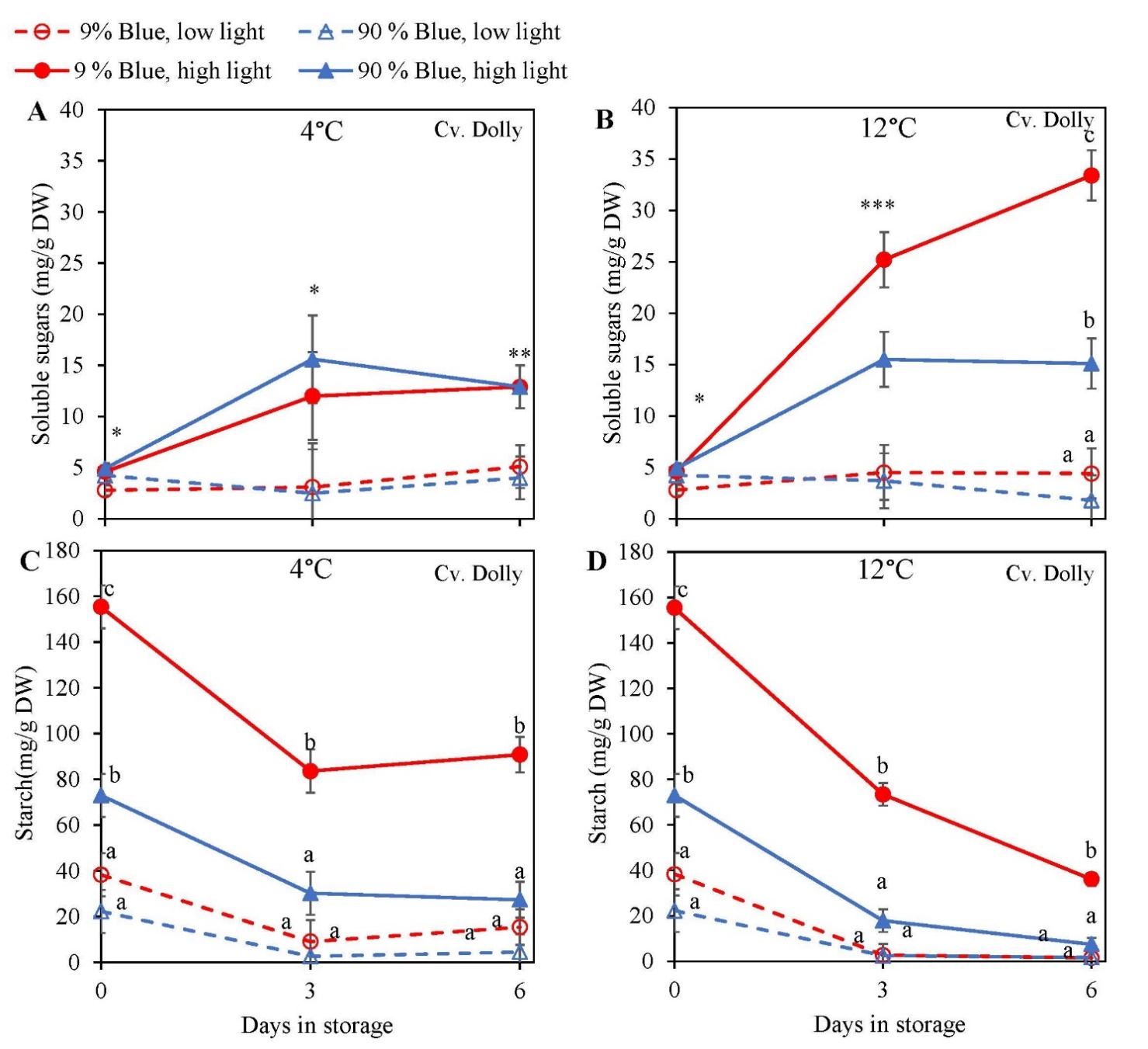


**Figure S3.** Changes in metabolite levels during postharvest storage at 4 (panels A,C) and 12° C (panels B,D) in basil cv Dolly. Plants were grown under red-white light (PPFD of 200 µmol m^-2^ s^-1^, 9% blue). The last five days before harvest plants were exposed to different EOP blue light ratios 9 % or 90 % at low light (100 µmol m^-2^ s^-1^) (open symbols) or high light (300 µmol m^-2^ s^-1^) (closed symbols). A and B. soluble sugars (sum of glucose, fructose and sucrose), C and D, change starch. All values are expressed per g DW in leaves. Data are means of 3 blocks (n=3) with 4 replicate plants per block. Standard errors of means are shown as error bars. If no interaction was found but only the main effects were significant the indicated with *p*-values; *p<0.05 *, p<0.01 **, p<0.001 **** are depicted with either a blue (percentage of blue light) or black asterisk (PPFD). Letters indicate and interactive effect between the two main effects (percentage of blue light and PPFD), (Exp. 2).


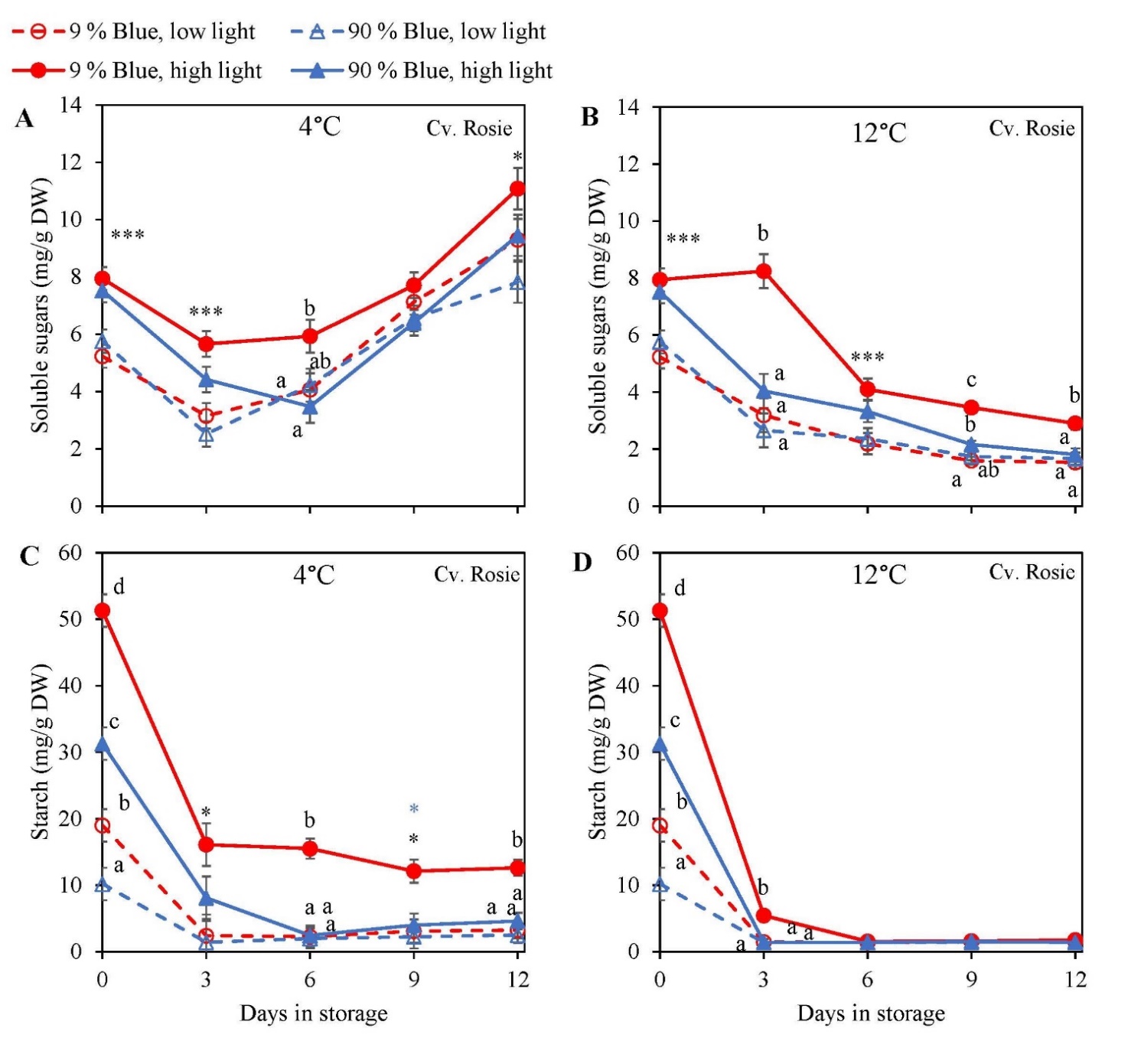


**Figure S4.** Changes in metabolite levels during postharvest storage at 4 (panels A,C) and 12° C (panels B,D) in basil cv Rosie. Plants were grown under red-white light (PPFD of 200 µmol m^-2^ s^-1^, 9% blue). The last five days before harvest plants were exposed to different EOP blue light ratios 9 % or 90 % at low light (100 µmol m^-2^ s^-1^) (open symbols) or high light (300 µmol m^-2^ s^-1^) (closed symbols). A and B. soluble sugars (sum of glucose, fructose and sucrose), C and D, change starch. All values are expressed per g DW in leaves. Data are means of 4 blocks (n=4) with 4 replicate plants per block. Standard errors of means are shown as error bars. If no interaction was found but only the main effects were significant the indicated with *p*-values; *p<0.05 *, p<0.01 **, p<0.001 **** are depicted with either a blue (percentage of blue light) or black asterisk (PPFD). Letters indicate and interactive effect between the two main effects (percentage of blue light and PPFD), (Exp. 2).

**
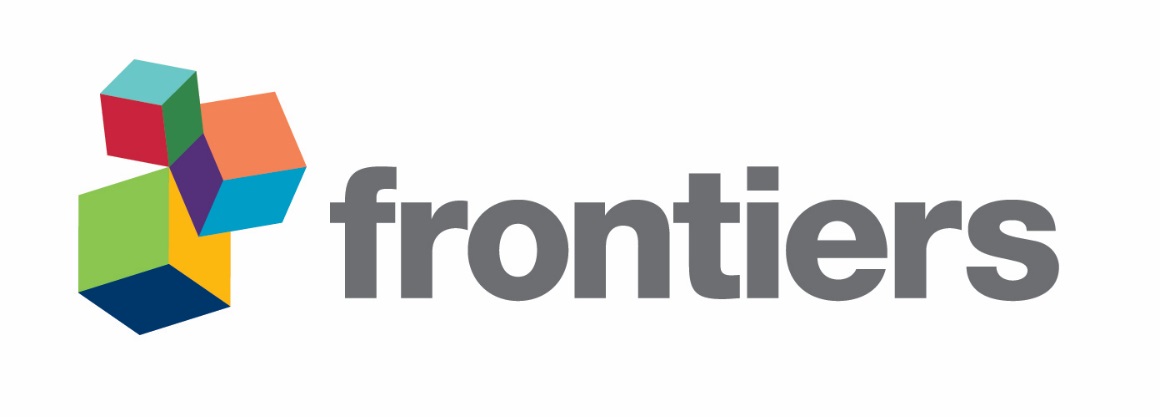
**
